# Label-free TIMING: an efficient, reliable and scalable AI workflow for automated profiling of cell-cell interaction behaviors in nanowell arrays

**DOI:** 10.3389/fbinf.2026.1711797

**Published:** 2026-02-12

**Authors:** Anuj S. Todkar, Shyam Reddy Kotha, Lin Bai, Saikiran Mandula, Hannah B. Wilson, Daniel D. Meyer, Rebecca Berdeaux, Badrinath Roysam, Navin Varadarajan

**Affiliations:** 1 William A. Brookshire Department of Chemical and Biomolecular Engineering, University of Houston, Houston, TX, United States; 2 CellChorus Inc., Houston, TX, United States; 3 Department of Electrical and Computer Engineering, University of Houston, Houston, TX, United States

**Keywords:** biological image analysis, label free assay, cell segmentation and classification, cell tracking and tagging, computer vision, dynamic single-cell analysis, time-lapse imaging, immunotherapy

## Abstract

Time-lapse imaging microscopy in nanowell grids (TIMING) is an integrated method for dynamic profiling of live immune–target cell interactions at single-cell resolution with broad applications and impact in immunology, immunotherapy and infectious diseases. Notwithstanding these applications, the current TIMING workflows necessitate fluorescent labeling of cells for automated image analysis operations including cell classification, segmentation, and tracking. Leveraging advances in computer vision methods for label-free phase contrast time-lapse microscopy and constraints specific to TIMING, especially spatial confinement of interacting cell cohorts in an array of nanoliter-capacity wells (nanowells); and temporal consistency, we show that TIMING analysis can now be performed in a fully label-free manner, with an accuracy comparable to the fluorescence-based TIMING. The proposed label-free TIMING (LF-TIMING) method offers reduced cellular phototoxicity and fluorescence photobleaching, reduced dye-induced artifacts that can interfere with physiological accuracy and enhanced live-cell imaging duration by eliminating reliance on fluorescent labels. Importantly, it expands the versatility of TIMING by enabling direct profiling of precious patient derived cells without the need for labeling while also freeing up fluorescence channels for investigating experimental structural or functional reporters, thus extending the molecular/subcellular features that can be profiled.

## 1 Background and introduction

**FIGURE 1:**
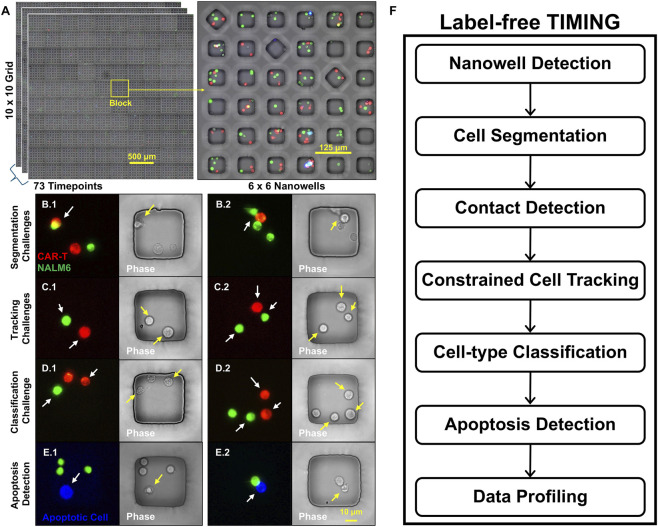
Illustrating the challenge of label-free (LF) TIMING analysis, and the opportunity to achieve high yield from exploiting the cell containment in nanoliter capacity wells (nanowells). **(A)** Part of a TIMING chip containing a 60 × 60 array of nanowells arranged in blocks of 6 × 6. Inset, an enlarged view of one block. **(B.1, B.2)** Representative examples showing the challenges in LF analysis due to cells in contact **(B.1)** and overlap **(B.2)** making boundaries ambiguous (yellow arrows), whereas the same cells have clear separable boundaries in the fluorescence images (white arrows). **(C.1, C.2)** Representative examples showing LF cell tracking challenges. In the phase channel, cell morphologies and appearance are challenging to distinguish while changing focus, and cells moving along well walls can cause identity swap errors during contact/occlusion (yellow arrows). The fluorescent channel provides stable appearance cues, making frame-to-frame linking straightforward (white arrows). **(D.1, D.2)** Cell type classification challenges. Distinguishing effector vs. target cells from solely phase-contrast is difficult because morphology provides subtle or poor cues (yellow arrows). Fluorescent labels can help distinguish cell types. **(E.1, E.2)** Death detection. The Annexin V marker (white arrows) can indicate the time of death, whereas phase-only cues are more subtle. (yellow arrow). **(F)** The proposed label free analysis workflow is made possible by overcoming the challenges at each step, and exploiting the nanowell containment constraint that guarantees constant cell counts over time barring mitosis/apoptosis.

Time-lapse imaging in nanowell grids (TIMING) is an integrated method for dynamic profiling of immune–target cell interactions at single-cell resolution across tens of thousands of spatially confined cells (Liadi et al., 2013; Lu et al., 2019; Merouane et al., 2015). In TIMING, cells of interest are co-cultured on chips containing arrays of thousands of nanoliter capacity wells (“nanowells”) and imaged at 3–7 min intervals over hours, yielding a large array of time-lapse video recordings (Figure 1A). By simultaneously tracking cell motility, contact dynamics, and fate outcomes, TIMING has played a pivotal role in uncovering functional heterogeneity in immune effector populations and mechanistic correlates of cytotoxicity in cancer immunotherapy (Li et al., 2022; Liadi et al., 2013; Rezvan et al., 2024; Romain et al., 2014; Sytsma et al., 2025) and evaluating cytotoxic lymphocytes in infectious diseases (Fathi et al., 2022).

While the TIMING assay meets critical needs, it has a limitation–it relies on multi-channel fluorescent imaging for cells, cell-type markers, and cell-status reporters to achieve reliably automated image analysis. Our prior work using these fluorescent images produced an automated software pipeline for analyzing TIMING video arrays to detect and segment cells and subcellular compartments, identify cell types, track movements, record contacts, detect cell death, and quantify intracellular proteins to generate a comprehensive readout of individual cell behaviors and activity while preserving cell viability (Lu et al., 2019). Unfortunately, there is a disadvantage to fluorescence imaging in the context of high-throughput time-lapse systems. Even the best-available widefield high-throughput time-lapse imaging instrumentation can only provide four to five fluorescent channels of which 2–3 channels must be devoted to labeling effector and target cells, leaving only one to two channels to study proteins of investigational interest. The fluorescent dyes also increase phototoxicity and restrict the duration of imaging (Merouane et al., 2015; Sadeghi et al., 2022). Finally, photobleaching and dye-induced artifacts can interfere with physiological accuracy. While phase-contrast video is routinely captured in TIMING, it has remained underutilized because traditional image analysis methods struggle with its low contrast and artifact-rich content (Bise et al., 2011; Rogers et al., 2007).

Much progress has been made with label-free (phase-contrast or brightfield) microscopy image analysis for individual tasks, *e.g.*, cell segmentation (Ker et al., 2018), selective focus restoration and the detection of apoptotic bodies (Wu et al., 2023; Wu et al., 2024), cell death detection (Huh et al., 2012), and mitosis detection (Huh et al., 2011). However, realizing a complete label-free TIMING analysis pipeline has remained a challenge due to the imaging scale and yield requirements (the percentage of nanowells that are correctly analyzed). As illustrated in Figure 1, wide-field label-free microscopy produces images of variable quality with uneven illumination, halo artifacts, and rapid morphological changes. This feature of label-free imaging makes accurate segmentation challenging, especially at scale. This problem is accentuated in one of the core functions of TIMING platform: the ability to detect cell-cell contacts reliably (Merouane et al., 2015). In the context of immunotherapy, this is the formation of a synapse between an immune cell and its target cell. The availability of fluorescent labeling for cell bodies and cell type markers made identification of cell boundaries and thus overlap a tractable problem (Merouane et al., 2015). With label-free images, unclear cell boundaries in the contact region make accurate segmentation and contact detection challenging (Figure 1B). Second, TIMING datasets often use lymphocytes as both effector cells and target cells (tumor cells). This presents a challenge to cell classification and tracking due to the visual similarities of the cell types (Figures 1C,D). Third, unlike label-free microscopy tracking adherent cells, the immune cells tracked in TIMING are motile (5–10 μm/min) and under drastic changes in morphology. Along with the acquisition rate (frame rate of 3–7 min), this presents a more difficult computer vision challenge compared to widely used video analysis methods (24 frames per second) that expect incremental changes from one frame to the next and exploit color and textural cues in color (RGB) video. The goal of transitioning to a complete label-free TIMING analysis pipeline requires that all the tasks detailed above that are currently accomplished using fluorescent dyes including cell segmentation, cell-type identification, movement tracking, and apoptosis detection (Figure 1E) must be executed using label-free videos alone, in high-throughput, with sufficient yield, reliability and efficiency, with an accuracy comparable to the fluorescence-based system. A fully label-free workflow can reduce phototoxicity and enhance imaging duration by eliminating reliance on fluorescent labels, and free up fluorescence channels for experimental structural or functional reporters, thus extending the molecular/subcellular features that can be profiled using TIMING (Cireşan et al., 2013).

In this paper, we introduce an integrated, end-to-end, label-free analysis framework for label-free TIMING (LF-TIMING) that leverages advanced deep neural networks (DNNs), and constraints specific to TIMING, especially the spatial confinement of cells in nanowells, to accomplish accurate cell segmentation, tracking, classification, and apoptosis detection directly from phase-contrast microscopy images, at an accuracy comparable to fluorescence based analysis. We are also encouraged by advances in recent generalist and promptable models, such as Cellpose 2.0, Omnipose, and SAM/SAM2, but as we illustrate, these models are not sufficiently accurate for routine TIMING analyses despite training (Cutler et al., 2022; Kirillov et al., 2020; Pachitariu and Stringer, 2022; Stringer et al., 2021). To build the ground truth datasets required to train these models, we utilized an iterative bootstrap strategy that grows the training corpus efficiently by leveraging existing cell-segmentation tools for handling the vast majority of nanowells, efficiently isolating the nanowell videos that are suspected/found to be analyzed erroneously using available analytics and machine learning tools, thereby focusing the human supervision and proofreading on this smaller subset of cases. Using this strategy, we trained our models on progressively larger corpuses of training data.

## 2 Materials and methods

### 2.1 Cell culturing and imaging

Single-cell cytotoxicity assays were performed as described previously (Liadi et al., 2013; Merouane et al., 2015). The nanowell arrays used were a combination of those fabricated as described previously and briefly fixed on a 50 mm glass-bottom Petri dish (Ted Pella) (Liadi et al., 2013; Merouane et al., 2015) and with nanowell arrays produced for commercial scale (CellChorus Inc., Houston, TX). CD19-CAR T cells (effector) and NALM6 cells (target) were labeled with 2 µM PKH 67 and 2 µM PKH 26, respectively. The effectors and the targets were loaded sequentially onto nanowell arrays at a concentration of 10^6^ cells per mL, and the entire nanowell array was incubated in complete media containing Annexin V conjugated with Alexa Fluor 647 (Invitrogen) at 37 °C and 5% CO_2_. The cells were imaged using a Carl Zeiss Axio Observer fitted with a Hamamatsu sCMOS camera using a 20 ×0.8 NA objective for 6 hours at 5-min intervals.

Because each raw field of view contains a large portion of the nanowell array, we convert array-level frames into per-well movies before downstream analysis. We localize individual wells using a Faster R-CNN detector (Girshick, 2015) trained to identify well rims and to classify wells as occupied versus empty. At inference, detections on the first frame yield bounding boxes that are refined with non-maximum suppression and fit to the expected lattice; the resulting grid is then propagated to subsequent frames and adjusted for small rigid shifts. For each localized well we extract a centered square crop at every time point, discard empty or partial-rim wells, and assign a persistent well identifier so that predictions can be mapped back to the parent video. This cropping step standardizes spatial context, prevents cross-well contamination, and enables efficient parallel processing in the subsequent segmentation, tracking, and classification modules.

### 2.2 Label-free overlapping cell segmentation network (LOCSNET)

Label-free segmentation presents significant challenges because label-free images can exhibit low contrast, varying illumination, halo artifacts, and frequent cell overlaps that degrade classical methods such as thresholding, watershed, and seed-based region growing (Rogers et al., 2007). Even strong semantic models like U-Net and VGG-based variants segment pixels well yet cannot reliably separate touching instances in dense nanowells where single-cell analysis is required (Ronneberger et al., 2015; Wang, 2019). In this regard, the widely used Mask R-CNN (He et al., 2017; Lin et al., 2017) method combining region proposals with multi-scale features for detecting and segmenting individual objects is a starting point. The first stage uses a convolutional backbone with a Feature Pyramid Network to extract multi-scale features and a Region Proposal Network to propose regions of interest. The second stage applies parallel heads for bounding-box regression, classification, and pixel-level masks. This two-stage design is advantageous in our setting because proposal-based instance models help maintain identity separation in crowded scenes, and pyramid features preserve small, low-contrast morphology typical of TIMING phase-contrast data. Despite its robustness, the Mask R-CNN can underperform when cells strongly overlap or partially occlude one another, which motivates the occlusion-aware extensions described next. For this, we introduce LOCSNET, which explicitly models the cell overlap pattern to reduce merge and split errors. We retain the two-stage pipeline and the baseline loss of the Mask-RCNN network,

$$L_{\text{MRCNN}\;}={\lambda_{1}L}_{\text{reg}}+{\lambda_{2}L}_{\text{cls}}+\lambda_{3}L_{\text{mask}\;}$$ (1)

where $L_{\text{reg}}$ represents the smooth-L_1_ loss for bounding box regression, $L_{\text{cls}}$ is the cross-entropy loss for classification, $L_{\text{mask}}$ is the pixel-wise cross-entropy loss for segmentation in stage two, and $\lambda_{1},\lambda_{2}$ , $\lambda_{3}$ are loss weights that are set by the training process.

Traditional segmentation approaches focus on the visible segments of overlapping objects, largely due to the inherent challenge of representing occluded areas where they are not directly observable. To overcome this, we introduce two additional mask heads to capture the overlapping and complement parts of the objects, alongside the standard whole object representation. We denote these mask heads $h_{c}$ , $h_{w},h_{o,}$ for the complement, whole mask, and overlap mask heads, respectively. The inputs of $h_{c}$ and $h_{o}$ for the $j^{th}$ cell in the $i^{th}$ image are $f_{i,j}^{\text{ROI}}$ , and the output feature maps of the $h_{c}$ and $h_{o}$ from the third convolution layer are added with $f_{i,j}^{\text{ROI}}$ to obtain the feature map $f_{i,j}^{\text{added}}$ for the whole mask head. The loss functions for the mask heads are formulated as follows:

$$L_{\text{mask}\;}=\frac{1}{KN}\;\sum_{i=1}^{K}\sum_{j=1}^{N}\left(L_{w}+L_{c}+L_{o}\right)$$ (2)

$$L_{w}{=L}_{\text{ce}}\left(h_{w}\left(f_{i,j}^{\text{added}}\right),e_{i,j}^{w}\right)$$ (3)

$$L_{o}{=L}_{\text{ce}}\left(h_{o}\left(f_{i,j}^{\text{ROI}}\right),e_{i,j}^{o}\right)$$ (4)

$$L_{c}{=L}_{\text{ce}}\left(h_{c}\left(f_{i,j}^{\text{ROI}}\right),e_{i,j}^{c}\right)$$ (5)

where $L_{\text{mask}\;}$ is the total loss for all samples, K is the total number of image frames, N is the number of cells, $e_{i,j}^{o}$ and $e_{i,j}^{c}$ represent the overlapping and complement parts of the cell mask $e_{i,j}$ respectively, and $L_{\text{ce}}$ is the cross-entropy loss function. Our model is trained in an end-to-end manner using the following composite loss function:

$$L_{\text{LOCSNET}\;}={\lambda_{1}L}_{\text{reg}}+\lambda_{2}L_{\text{cls}}+\lambda_{3}L_{\text{mask}\;}$$ (6)

**FIGURE 2:**
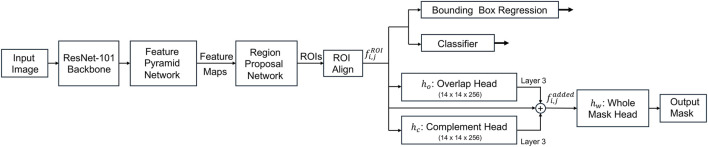
Block diagram depicting the LOCSNET network architecture as an extension of the Mask-RCNN network with the addition of the overlap and complement heads with the same head architecture as the Mask-RCNN. The features at Layer three of the added mask heads and $f_{i,j}^{ROI}$ are combined into $f_{i,j}^{added}$ from which the output mask is computed.

While $L_{\text{LOCSNET}\;}$ ​ shares the same structure as the baseline $L_{\text{MRCNN}\;}$ (Equation 1), the $L_{\text{mask}\;}$ ​ term here is critically redefined as the composite loss from our three new mask heads (described in Equations 2–5), rather than the original single-head mask loss (Figure 2, Equation 6). LOCSNET reduces ambiguity at cell–cell contacts and improves instance separation without sacrificing detection recall. This approach is consistent with recent occlusion-aware and boundary-aware advances in instance segmentation that show gains in dense and overlapping regions (Hayder et al., 2017; Jiang et al., 2023; Ke et al., 2021; Kirillov et al., 2020; Wu et al., 2023), and it is well aligned with TIMING’s requirement for accurate per-cell masks in confined wells.

An input video frame is first processed by a standard Mask-RCNN backbone to generate a feature map from which a region of interest is proposed. This region is processed by three parallel mask heads: (a) the overlap mask head predicts only the pixels where 2 cells overlap, (b) the complement mask head predicts the visible, non-overlapping parts of the cells, and (c) the whole mask head predicts the complete boundary for each individual cell.

### 2.3 Globally optimal containment constrained cell tracking for nanowell videos

Reliable tracking of individual cells over time, on a large scale, is essential for quantifying contact events, migration dynamics, and apoptosis in nanowell videos. In TIMING, tracking errors are consequential, since a single frame-to-frame tracking error can invalidate measurements extracted from an entire nanowell video, greatly reducing the assay yield. As noted earlier, the low frame rate in TIMING implies that frame-to-frame cell movements can be large, so video tracking methods are not usable. In addition, cell segmentations can fail, cells can overlap, motion patterns can shift abruptly, and cell boundaries can have poor contrast. We describe the following three-step strategy to overcome these challenges.

Step 1: Generation of an initial frame-to-frame tracking assignment matrix: We exploit the fact that the physical confinement of cells within nanowells effectively guarantees a constant cell count in each nanowell over time, unless there is segmentation error (missed cell, false detection), or a (rare) mitotic event. With this in mind, we start by identifying the best- N matches between every pair of frames t and t +1, where N is the cell count based on the following pair-wise assignment costs (Equation 7):

$$C_{i,j}^{\left(t\right)}={\left\|p_{j}^{\left(t+1\right)}-p_{i}^{\left(t\right)}\right\|}^{2}+\lambda_{\text{vel}}{\left\|{\hat{v}}_{i}-v_{\text{ij}}\right\|}^{2}{-\beta }_{\text{IoU}}\cdot \text{IoU}\left(M_{i}^{\left(t\right)},M_{j}^{\left(t+1\right)}\right)+\lambda_{\text{switch}}\cdot I_{\text{switch}}$$ (7)

where $p_{i}^{\left(t\right)}$ and $p_{j}^{\left(t+1\right)}$ ​ are cell centroids, $v_{\text{ij}}$ *=* ${\;p}_{j}^{\left(t+1\right)}-p_{i}^{\left(t\right)}$ are observed cell displacements, ${\hat{v}}_{i}$ is a short-horizon cell velocity estimate, and IoU (⋅) measures the shape similarity of cell masks $M_{i}^{\left(t\right)}$ and $M_{j\left(t+1\right)}$ . The third term is designed to penalize inconsistent matches that may occur following a cell contact event. Specifically, when a pair of cells separate after a contact event, it is possible for their identities to be switched incorrectly. With this in mind, $I_{\text{switch}}$ is a binary variable that is set to one whenever 2 cells are in contact, and $\lambda_{\text{switch}}$ is an empirically fixed penalty (typ. ∼100). We minimize $C_{i,j}^{\left(t\right)}$ using the Hungarian algorithm, and this is computationally acceptable since *N* is small (<5). We compute links in the forward and reverse directions to expose any inconsistencies (Jaqaman et al., 2008; Kuhn, 1955; Tinevez et al., 2017). The resulting assignments are accurate for most frames for which the cell counts are equal. For the remaining frames, we use the strategy described next.

Step 2: Prediction of Missed Cell Segmentations: In this step, we re-analyze the frame-to-frame cell tracks from Step 1 over entire nanowell videos to detect frames for which the cell count is < N , and computationally predict the locations of any missed cells by solving a convex optimization problem based on the premise that cell velocities are consistent over each nanowell video. For each cell i , we define cell centroid trajectories $\left\{p_{i}^{t}:t=1,2,..\;K\right\}$ across all frames and minimize the sum of squared velocities, subject to equality constraints that anchor the trajectory to the measured centroids at frames with detections. This provides an interpolated centroid value at each frame and fills in short gaps. However, this does not address the problem of tracking through occlusions and cell contacts. This is addressed in Step 3 below.
Step 3: Global Optimization based Reconciliation of Tracking Assignments: In this step, we reconcile the forward and reverse assignments and refine the cell trajectories using a global optimization algorithm. For this, we formulate a convex optimization that balances smoothness and fidelity to the provisional assignments from Step 2, by minimizing the loss (Equation 8):

$$L=\lambda_{\text{smooth}}\sum_{t}{\left\|\Delta v_{t}\right\|}^{2}+\alpha_{\text{acc}}\sum_{t}\left\|\Delta^{2}p_{t}\right\|+\alpha_{\text{fid}}\sum_{t}{\left\|p_{t}-{\bar{p}}_{t}\right\|}^{2}$$ (8)

where $\Delta p_{t}=p_{t+1\;}-p_{t\;},$ $\Delta v_{t}=p_{t+1\;}-p_{t},\Delta^{2}p_{t}=v_{t\;}-v_{t+1\;}$ and ${\bar{p}}_{t}$ denotes the refined cell centroid from Step 2. For this quadratic optimization, we used the CVXPY/OSQP open source solver for optimizing all tracks over each full sequence. This produces trajectories that are spatially consistent and temporally smooth, while reducing jitter and identity flips following cell occlusions. This sequence-level reconciliation follows global association strategies that stabilize tracks in crowded nanowells.

To validate the tracking framework, we applied it to a large collection of segmented TIMING videos. Human annotators manually corrected track irregularities, including missed detections and identity mismatches, to generate a set of curated ground-truth trajectories. This curated dataset was used exclusively as a reference for validation, not for training. We compared the tracks generated by our unsupervised algorithm against this reference set using multi-object tracking metrics such as MOTA (Multiple Object Tracking Accuracy), identity switches, and per-frame matching costs. These evaluations demonstrate that our framework is accurate, robust, and well-suited to the high-throughput, label-free tracking demands of TIMING data.

### 2.4 Temporally consistent label-free cell-type identification

Cell type identification from label-free data is challenging since it relies upon morphological and appearance cues that are subtle and not nearly as robust as fluorescent markers (Figure 1). In this regard, using a CNN classifier is fraught with the challenge from inconsistent labeling from one frame to the next. We address this using a hybrid model that combines convolutional neural networks (CNNs) with long short-term memory (LSTM) units. CNNs are strong spatial feature extractors with state-of-the-art performance across vision benchmarks, and they effectively capture texture and morphology in microscopy images (Krizhevsky et al., 2017; Ronneberger et al., 2015). However, CNNs alone are not sufficient for modeling temporal dependencies that are critical for distinguishing subtle phenotypes such as pre-apoptotic versus apoptotic cells. LSTMs, a class of recurrent neural networks, address this gap by maintaining memory over sequences and learning patterns that evolve over time.

Our CNN-LSTM architecture comprises three components. First, we use a CNN backbone (ResNet-50) adapted to accept four-channel inputs: the phase-contrast image replicated into three channels plus a binary mask channel. While ResNet-50 was used for the final model, our evaluation also compared ResNet-18 and ResNet-34. The selection of these backbones was strategic, balancing model complexity with representation capacity. ResNet architectures were chosen due to their proven efficacy in feature extraction for visual recognition tasks, facilitating efficient spatial feature learning from sequential image patches (He et al., 2017). The CNN encodes spatial features of each cell at every time point into a compact embedding. Second, these embeddings are concatenated with motion descriptors derived from the tracked centroid (for example, speed, direction, and short-horizon acceleration), which provide kinematic context. Third, the per-frame feature sequence is passed to an LSTM that models temporal dynamics and outputs a hidden state summarizing the results over the entire video track. A fully connected layer maps this representation to a binary label (for example, effector versus target, or alive versus apoptotic). We retain pretrained CNN weights to leverage general image priors and we fine-tune the temporal module and classification head. We use dropout and stratified sampling to reduce overfitting. Prior work has validated CNN–LSTM designs for video-based microscopy tasks including mitosis detection, fate prediction, and cell tracking (Buggenthin et al., 2017; Cireşan et al., 2013; Su et al., 2012).

For all sequence-level classification tasks in this work (cell-type identification in TIMING wells and apoptosis detection in Section 2.5), we report accuracy, sensitivity (recall), specificity, positive and negative predictive values (PPV, NPV), and the area under the receiver operating characteristic curve (AUROC). Accuracy provides an overall summary, whereas sensitivity and specificity separately quantify missed true effector/target events versus spurious detections. PPV and NPV are important in our setting because some nanowell cohorts are moderately imbalanced (for example, more non-killing than killing interactions), and these metrics capture how often positive and negative predictions are correct. AUROC offers a threshold-independent summary of discrimination that remains robust under such class-imbalance. Together, this set of metrics provides a comprehensive and interpretable characterization of classifier performance that is consistent with standard practice in diagnostic and microscopy-based classification studies.

One key application of our CNN–LSTM model is label-free classification of immune effector and target cells in TIMING videos. Traditionally, cell types are identified using fluorescent dyes, however, such markers restrict imaging throughput, increase phototoxicity, and limit multiplexing capacity. To overcome these limitations, we propose a data-driven approach that predicts cell type directly from phase-contrast videos notwithstanding the weak cues (Möckl et al., 2020; Ounkomol et al., 2018). To train the CNN, we first generate ground truth labels by aligning each cell’s tracked centroid and segmentation mask to its fluorescent channel intensity. Cells consistently positive for the effector or target label across multiple frames are used as anchors. Then, corresponding sequences of phase-contrast patches and motion features are extracted and paired with their binary identity label. Our training set includes thousands of such segments spanning dozens of videos and cell lines, ensuring sufficient representation of variability in size, shape, and movement. To achieve robust cell classification, we exploit temporal continuity by framing the problem as a sequence-level binary classification rather than a frame-level classification. Given a sequence of image patches and motion vectors​, the model predicts a single label for the entire track. The use of LSTM enables the model to capture temporal patterns such as migration style or stable polarization, which are difficult to encode via static features.

### 2.5 Temporally consistent label-free apoptosis detection

Apoptosis, or programmed cell death, is a critical endpoint in immune cell–target cell interactions and is traditionally detected using biochemical markers like Annexin V, which binds to phosphatidylserine exposed on the outer membrane of apoptotic cells (Merouane et al., 2015). While highly specific, fluorescent apoptosis detection limits imaging duration due to photobleaching and phototoxicity. A label-free alternative, using phase-contrast microscopy, allows for long-term monitoring but requires models that can capture subtle and time-evolving morphological and appearance changes associated with apoptosis, *e.g.*, blebbing.

For this, we trained our CNN–LSTM classifier to detect apoptosis based solely on phase-contrast and mask inputs. For the training, we use cells that were confidently labeled apoptotic using the Annexin V marker. A cell is marked as alive until the first fluorescent-positive frame, and apoptotic from that point onward. This yields a temporally ordered sequence of labeled cell bounding boxes and the corresponding morphological features (e.g., area, eccentricity), that we use to train the model. The LSTM is particularly valuable for stable apoptosis detection due to its irreversible nature: once a cell enters the apoptotic state, it does not revert. To capture this monotonicity, we add a regularization term to the loss function that penalizes downward fluctuations in the predicted apoptotic probability over time. This is inspired by prior work in modeling irreversible transitions in cell biology (Ismail Fawaz et al., 2019). The final output is a frame-wise prediction sequence, representing the likelihood of apoptosis at each time point. Our label-free classifier achieves high frame-level accuracy (AUROC >0.92) and precisely pinpoints apoptotic onset in most target cells. Compared to frame-by-frame CNN classifiers, the temporal model yields smoother outputs and suppresses false positives caused by transient shape fluctuations. These findings support the feasibility of replacing fluorescent apoptosis markers with deep learning–based morphology analysis in long-term studies.

## 3 Experimental results

For a full label-free implementation of TIMING analysis, it is important that each of the core steps, including cell segmentation, cell type identification, tracking, and apoptosis detection be achievable without the benefit of fluorescent labels. Accordingly, we evaluated the proposed pipeline of algorithms on a corpus of randomly selected nanowell videos from multiple datasets consisting of CAR T cells (effectors) and NALM6 cells (targets). For all the tasks in this paper, we followed the same workflow: training/test set generation, ground truth annotation, model training, and performance. There is no model training step in the cell tracking step since it is unsupervised. On the other hand, in the cell detection, segmentation, and apoptosis classification experiments, we leveraged unsupervised algorithms and semi-automatic human-in-the-loop proof-reading to create a large corpus of >2,500 annotated nanowell videos.

### 3.1 Cell segmentation and contact detection

To evaluate the segmentation accuracy of our LOCSNET model, we compared its performance against two state-of-the-art baselines: Cellpose and SAM2, both fine-tuned on our dataset. We assessed cell segmentation accuracy using the mean Intersection-over-Union (IoU) metric on a held-out test set of 500 nanowell videos comprising over 50,000 frames and approximately 120,000 cell instances. The generalist methods struggled to segment phase-contrast frames even after they were fine-tuned on nanowell videos. Cellpose yielded an mIoU of 0.5 ± 0.3. This method depends on gradient-flow fields followed by thresholded mask extraction, and this makes its performance sensitive to probability and flow threshold settings (Pachitariu and Stringer, 2022; Stringer et al., 2021). In TIMING wells, phase halos and weak contrast at cell–cell interfaces make it difficult to set these sensitivity parameters, leading to missed detections, under-segmentation, and merges at contact. Next, the widely used SAM2 (Kirillov et al., 2020) model improved over Cellpose, yielding an mIoU of 0.8 ± 0.1. however, it is prompt and initialization dependent and often over-segmented touching cells in crowded nanowells because phase halos and subtle boundaries are interpreted as separate instances. A non-foundational Mask R-CNN model trained on the same 240 videos yielded a mIoU of 0.7 ± 0.2, better than Cellpose but worse than SAM2. It failed to efficiently learn overlaps (He et al., 2017), and this is understandable since its single binary mask head encourages one connected region per instance, and provides no explicit representation of the shared contact boundary or occlusion ordering, therefore over- and under-segmentation persist. The LOCSNET model (v1), when trained on the same 240-video corpus yielded an mIoU of 0.8 ± 0.2, outperforming Cellpose, SAM2, and MRCNN.

**FIGURE 3:**
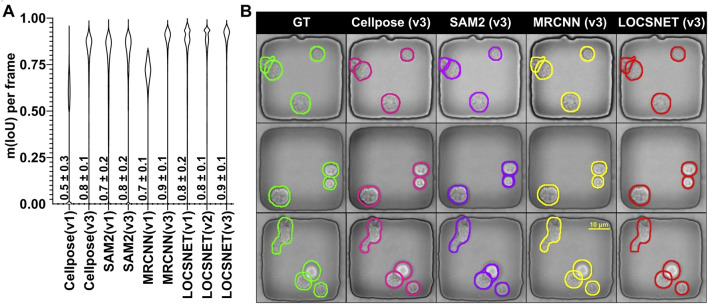
Performance of LOCSNET (v1, v2, v3), MRCNN (v1, v3), Cellpose (v1, v3), and SAM2(v1, v3) on TIMING data. **(A)** Violin plots comparing m (IoU) per frame across different segmentation models. **(B)** Qualitative visual comparison of segmentation results on three representative nanowell images. The columns show the Ground Truth (GT) and the outputs from Cellpose (v3), SAM2 (v3), MRCNN (v3), and our proposed LOCSNET (v3). The LOCSNET output more closely matches the ground truth, especially in cases of touching or overlapping cells where other methods tend to merge or miss instances.

The LOCSNET model effectively addressed the above mentioned failure modes by explicitly modeling three mask components, namely, the visible interior, the contact or overlap interface, and the full extent and by jointly optimizing them (Figure 2). Unlike the standard Mask R-CNN, which tends to merge contacting cells when trained on limited data, LOCSNET accurately resolves overlaps and occlusions even in small training regimes. When trained on only 240 videos, LOCSNET achieved a mean IoU of 0.8 ± 0.2, surpassing Cellpose, SAM2, and a non-foundational Mask R-CNN trained on the same corpus (0.7 ± 0.1). This advantage arises from its explicit representation of shared boundaries, which enhances data efficiency and robust generalization. Encouraged by this, we trained LOCSNET on progressively larger corpora, yielding mIoU values of 0.8 ± 0.1 (v2; 350 videos) and 0.9 ± 0.1 (v3; 1,000 videos) (Table 1). Although its performance converged with Mask R-CNN at larger scales, the overlap-aware design of LOCSNET remains advantageous when expanding to new cell types or experimental conditions where annotated data are limited. Importantly, LOCSNET can serve as a bootstrapping model for building new training sets, producing cleaner instance masks requiring minimal manual correction compared to conventional Mask R-CNN outputs, thereby reducing the burden of ground-truth curation and accelerating dataset growth. The narrowing violin plots and rising medians in Figure 3A underscore how architectural specialization, combined with domain-specific scaling, captures diverse morphologies, contact configurations, and illumination patterns more effectively than generalist or non-overlap models (Jiang et al., 2023; Ke et al., 2021).

**TABLE 1:** Per-frame segmentation accuracy (mIoU) for each model and training regime, with mean ± SD and 95% confidence intervals.

Model	Training videos	mIoU (mean ± SD)	95% CI of mean mIoU
Cellpose (v1)	240	0.5 ± 0.3	[0.48, 0.49]
Cellpose (v3)	1,000	0.8 ± 0.1	[0.82, 0.82]
SAM2 (v1)	240	0.7 ± 0.2	[0.74, 0.75]
SAM2 (v3)	1,000	0.8 ± 0.2	[0.78, 0.8]
MRCNN (v1)	240	0.7 ± 0.1	[0.71, 0.71]
MRCNN (v3)	1,000	0.9 ± 0.1	[0.89, 0.89]
LOCSNET (v1)	240	0.8 ± 0.2	[0.84, 0.84]
LOCSNET (v2)	350	0.8 ± 0.1	[0.85, 0.85]
LOCSNET (v3)	1,000	0.9 ± 0.1	[0.89, 0.90]

**FIGURE 4:**
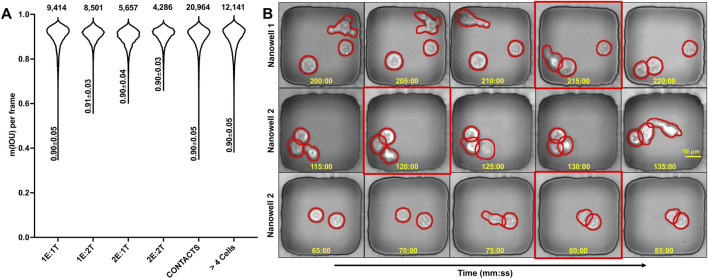
Performance of LOCSNETv3 is robust across effector-target combinations. **(A)** Violin plots showing the distribution of m (IoU) per frame across different experimental conditions. **(B)** Time-lapse sequences from three example nanowells showing successful segmentation (green outlines) over extended periods. The red boxes highlight frames with challenging cell interactions or morphological changes where the model maintains accurate segmentation.

To assess generalization, we stratified the cell segmentation performance by effector-to-target (E:T) cell counts, and the results are summarized in Figure 4. This notation refers to the number of cells present in the nanowell (e.g., 1 Effector:1 Target or 2 Effectors:2 Targets). LOCSNET v3 showed robust performance, with mIoU values for 1E:1T (0.90 ± 0.05; 95% CI [0.90, 0.90]), 1E:2T (0.91 ± 0.03; 95% CI [0.91, 0.91]), 2E:1T (0.90 ± 0.04; 95% CI [0.89, 0.90]), and 2E:2T (0.90 ± 0.03; 95% CI [0.90, 0.90]). The accuracy remained high in contact-rich nanowells (0.90 ± 0.05; 95% CI [0.90, 0.90]) and in crowded nanowells with more than 4 cells (0.90 ± 0.05; 95% CI [0.89, 0.90]). These results show that instance separation remains reliable even in crowded nanowells with higher cell overlaps and occlusions.

For contact detection, frame-wise predictions were compared with manual annotation across 192 videos. The row-normalized confusion matrix reports True Negatives (TN) of 97.7%, False Positives (FP) of 2.3%, False Negatives (FN) of 29.6%, and True Positives (TP) of 70.4%, yielding an overall label-free contact detection accuracy of 0.9, in line with the performance using fluorescence. Manual inspection of the results showed that many false negatives convert to true positives within the next one to two frames, consistent with small mask gaps or transient under-segmentation at the effector–target interface. In practice, this yields reliable contact onsets with a slight temporal lag when boundaries are uncertain.

### 3.2 Label-free nanowell containment-constrained cell tracking performance

To quantify tracking accuracy under representative TIMING conditions, we assembled a test set of 252 nanowells having different effector–target (E:T) ratios, frequent contacts, and occlusions. Performance was evaluated with Multiple Object Tracking Accuracy (MOTA) (Bernardin and Stiefelhagen, 2008; Chenouard et al., 2014; Ulman et al., 2017), defined as (Equation 9):

$$\text{MOTA}=1-\frac{\Sigma_{t}\left({FP}_{t}+{FN}_{t}+{IDSW}_{t}\right)}{\Sigma_{t}{GT}_{i}}$$ (9)

where ${FP}_{t}$ is the number of false-positive detections at frame t , ${FN}_{t}$ is the number of missed (false-negative) detections, ${IDSW}_{t}$ is the number of identity-switch events between frames one and t , and ${GT}_{i}$ is the number of ground-truth objects (cells) in the nanowell. MOTA was selected for its ability to simultaneously penalize segmentation inaccuracies (missed or extra masks) and identity assignment errors (identity switches), thereby providing a comprehensive, intuitive metric aligned with best practices in the cell tracking literature (Chenouard et al., 2014; Ulman et al., 2017). This approach is particularly relevant under confined nanowell conditions, where even brief identity misassignments significantly bias downstream analyses of cell-cell interactions (Wu et al., 2023).

**FIGURE 5:**
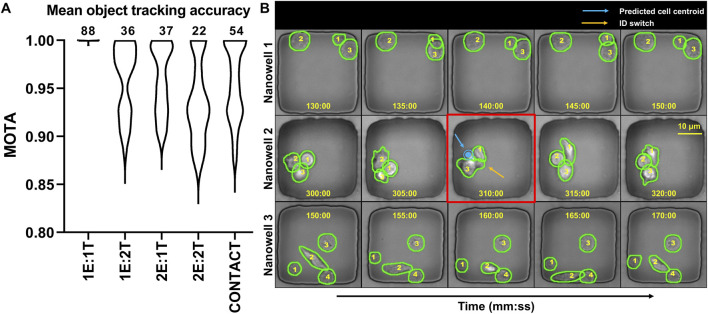
Tracking accuracy of our 3-stage label-free nanowell containment constrained cell tracker. **(A)** Mean label-free object tracking accuracy (MOTA) for 252 nanowells. The nanowell numbers for each cohort are indicated above the violin plots. **(B)** Sample Time-lapse sequences from three nanowells to illustrate the performance of the cell tracking algorithm. Each cell is assigned a persistent numerical ID (e.g., 1, 2, 3) and is outlined by its segmentation mask. The red boxes highlight frames with challenging tracking events.

Figure 5 summarizes the label-free tracking performance across 252 wells. In the simplest configuration (1E:1T, n=88 ), mean MOTA was 1.00 ± 0.00 (95% CI [1.00, 1.00]), indicating near-perfect identity maintenance. Introducing a third cell (1E:2T, n=36 ; 2E:1T, n=37 ) resulted in a minor reduction to 0.96 ± 0.04 (95% CI [0.95, 0.97]) and 0.96 ± 0.03 (95% CI [0.95, 0.97]), respectively. In the most crowded condition (2E:2T, n=22 ), the performance remained high at 0.94 ± 0.04 (95% CI [0.92, 0.96]). Taken together, these label-free results show that the accuracy degrades gracefully with density and sustained contact yet remains ≥0.94 across E:T ratios, also in line with the performance observed with fluorescence data.

We attribute this robustness to the three-step tracking design described earlier: first, a one-to-one Hungarian linker that combines spatial proximity, short-horizon motion consistency, and mask overlap (IoU) in the linking cost; second, a convex minimum-velocity interpolation that fills brief gaps while anchoring the trajectory exactly at frames with detections; and third, global trajectory refinement that balances smoothness with fidelity to the provisional links to suppress jitter and short identity glitches near transient occlusions. Optimizing trajectories at the sequence level (rather than greedily frame-to-frame) reduces the accumulation of local errors and stabilizes tracks around contacts.

Because contact-rich wells are the most challenging, we further analyzed nanowells with frequent and prolonged overlaps. For brief occlusions (one to two frames) and intermittent merges, the combination of bidirectional linking, quadratic interpolation on the provisional tracks, and sequence-level refinement maintained high accuracy MOTA = 0.96 ± 0.04; 95% CI [0.94, 0.97]). However, extended occlusions (>2 frames) and persistent mask merging during tight conjugation can still induce identity switches and modestly increase variance, highlighting where future occlusion modeling and re-identification cues could help (Ulman et al., 2017; Wu et al., 2023).

Three specific failure modes were systematically identified and characterized to further understand tracking limitations. First, prolonged occlusions exceeding two consecutive frames introduced errors during linear interpolation, causing occasional mis-assignments in subsequent frames (Wu et al., 2023). Second, during intense cell-cell interactions, segmentation sometimes failed, either merging distinct cells or fragmenting a single cell. Once incorrect masks were established, global smoothing failed to rectify the resulting errors, inevitably leading to identity switches. Lastly, cells transiently moving beyond the imaging field and subsequently re-entering produced intermittent missed detections (Ulman et al., 2017). Although these scenarios minimally impacted overall performance, clearly identifying them provides a roadmap for targeted improvements. Integration of CVXPY-based global optimization significantly enhanced the accuracy, consistency, and reliability of cell tracking. Its capacity to efficiently handle global constraints and interpolate missing detections ensured robust performance across diverse and challenging scenarios, establishing the pipeline as a dependable tool for quantitative assessments in label-free, time-lapse microscopy studies (Chenouard et al., 2014; Wu et al., 2024). For perspective, the above-mentioned errors are challenging for human proofreaders as well.

### 3.3 Label-free cell-type identification

The CNN-LSTM cell classification model was trained using a rigorous 5-fold cross-validation, which significantly enhanced the model’s robustness and generalizability in distinguishing Effector (CAR T) cells from Target (NALM6) cells from label-free image sequences. This evaluation utilized expert-validated annotations generated through manual fluorescence-based labeling, providing high-confidence ground truth data (Wu et al., 2023).

**FIGURE 6:**
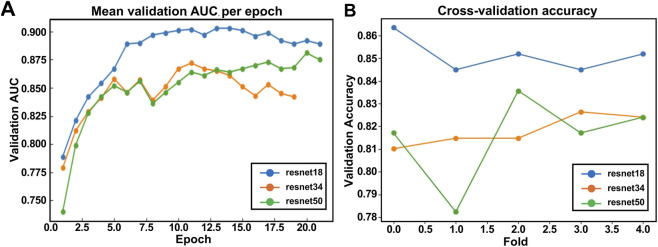
Backbone comparison for label-free temporally consistent cell-type classification. **(A)** A line plot showing the mean validation Area Under the Curve (AUC) over training epochs for the three ResNet backbones. **(B)** Plot showing the mean validation accuracy across five cross-validation folds for each backbone.

As depicted in Figure 6A, all models demonstrated rapid initial improvement in validation AUC, signifying efficient early-stage feature learning and effective differentiation between cell types. ResNet-18 consistently achieved the highest validation AUC, outperforming ResNet-34 and ResNet-50. This superior performance by the shallower ResNet-18 indicates its optimal capability to generalize well from limited labeled data while effectively mitigating overfitting. Figure 6B further illustrates model consistency across five cross-validation folds. ResNet-18 showed minimal variability in validation accuracy (86% ± 1%), highlighting its stable performance and reliability across diverse data subsets. In contrast, ResNet-34 and ResNet-50 exhibited more pronounced fluctuations (82% ± 2% and 82% ± 2%, respectively), suggesting increased sensitivity to training data heterogeneity and potential overfitting risks in deeper models (Simonyan et al., 2014) (Table 2).

**TABLE 2:** Performance comparison of ResNet backbones for label-free cell-type classification.

ResNet backbone	Mean validation accuracy (%)	Mean validation AUC	p-value using DeLong’s test
ResNet-18	86 ± 1	0.90 ± 0.01	-
ResNet-34	82 ± 2	0.86 ± 0.01	0.376
ResNet-50	82 ± 2	0.85 ± 0.01	0.024

The CNN-LSTM architecture was crucial for capturing both spatial and temporal information effectively. The CNN backbone extracts critical spatial texture and binary shape features from sequential patches, while the LSTM head integrates temporal dynamics, capturing motion patterns and cell trajectories crucial for distinguishing cell classes reliably. By combining motion descriptors (speed, direction, acceleration) with spatial features, the CNN-LSTM architecture significantly improved predictive accuracy compared to purely spatial or temporal models alone, highlighting the benefit of this integrated approach (Donahue et al., 2017). In our experiments, the CNN–LSTM outperformed frame-level CNN classifiers by a margin of approximately 5%–8% in sequence-level accuracy, with the largest gains observed in wells containing overlapping cells or subtle morphological differences. These results align with findings from prior video-based classification works in microscopy (Su et al., 2012; Zaritsky et al., 2021).

Specifically, the CNN-LSTM effectively differentiated between the distinct morphological and motility characteristics of CAR T and NALM6 cells. CAR T (effector) cells typically exhibit a smaller, rounded morphology with more pronounced membrane dynamics and motility, often characterized by rapid directional movements toward target cells. Conversely, NALM6 (target) cells generally present as slightly larger, more uniformly rounded, and less dynamically motile, with slower and less directed movements. These morphological differences, captured as spatial texture and shape features by the CNN backbone, provided foundational information for accurate classification. Meanwhile, the LSTM head leveraged temporal motion descriptors to exploit the substantial differences in movement patterns and trajectory dynamics between the 2 cell types, enabling precise temporal integration of these spatial cues.

The model training utilized the Cross-Entropy Loss function with label smoothing set to 0.1, effectively reducing overconfidence in model predictions and promoting generalization by preventing extreme logits. An AdamW optimizer with an initial learning rate of 3 $\times 10^{-5}$ and a cosine annealing scheduler further improved model convergence, maintaining stable updates and progressively fine-tuning weights across epochs. Heavy dropout (0.5) in the LSTM head and careful freezing of early CNN layers was applied strategically to reduce overfitting and promoting the model’s capacity to generalize across diverse data subsets. These hyperparameters collectively contributed to robust and reliable performance across different validation folds and challenging classification conditions.

**FIGURE 7:**
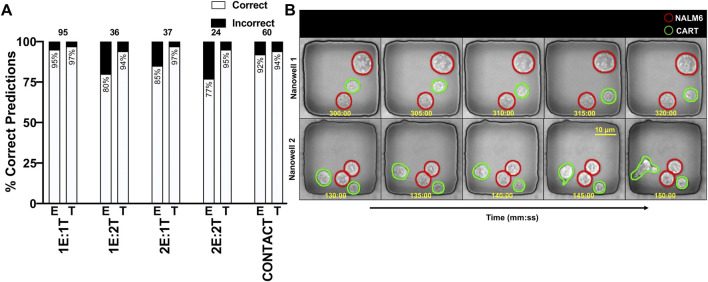
Label-free temporally consistent cell type identification across different effector to target (E:T) ratios. **(A)** A stacked bar chart showing the percentage of correct and incorrect classifications at the video level across different effector-to-target (E:T) ratios and in wells with cell contact. The model achieves high accuracy, correctly classifying the cell types in over 90% of videos in most conditions. Performance is slightly lower for effector cells in crowded wells (e.g., 2E:2T). **(B)** Qualitative examples from two nanowells showing the model’s predictions over time. Predicted NALM6 cells are circled in red, and predicted CAR T cells are circled in green, demonstrating successful identification based on morphology and motion alone.

We tested the label-free classifier on a panel of 252 manually annotated nanowell videos for every important effector(E):target(T) ratio, including videos having frames with contact (Figure 7). Initially, each video frame is processed by the overlap aware LOCSNET segmentation model to generate cell masks. These boxes are then linked across consecutive frames by the tracker, producing a unique trajectory, centroid series, and motion history for every cell. Only after this segmentation-and-tracking stage are the data forwarded to the classifier. For every tracked cell the pipeline crops the cell region and adjust the crop to 224 × 224-pixel phase-contrast patch centered on its centroid, appends a one-bit mask channel, and concatenates five motion descriptors that summarize speed, direction, and frame-to-frame acceleration. Sixty-four successive patches per trajectory are streamed through a ResNet-18 backbone to capture spatial texture and binary shape, after which a temporal LSTM head integrates the clip and issues a probability for CAR T effector versus NALM6 target. Using this purely phase-based information, the network achieves ≈88% accuracy for effectors and ≈95% for targets when benchmarked against manual annotations (Table 3). While it predicts with high accuracy in over 90% of nanowells, its confidence decreases when both contrast and motility cues are weak and are mostly notable in dim, slowly moving effectors residing in crowded 2E:2T wells (Figure 7). Expanding the training set with additional low-contrast or low-motility effector clips or applying focus restoration to existing sequences—should therefore be the most effective means of eliminating the residual 5%–15% error observed under these challenging conditions.

**TABLE 3:** Label-Free cell-type classification accuracy across different Effector (E) to Target (T) cell ratios.

Nanowell type	No. of videos	Total frames	Prediction accuracy (per video)			Sensitivity (recall)	Specificity	Precision (PPV)	NPV
			Effector	Target	Overall				
1 E:1 T	95	7,945	95%	97%	95.8% [92.9, 98.6]	94.7% [90.2, 99.2]	96.8% [93.3, 100.0]	96.8% [93.2, 100.0]	94.8% [90.4, 99.2]
1 E:2 T	36	3,067	81%	94%	89.8% [84.1, 95.5]	80.6% [67.6, 93.5]	94.4% [89.2, 99.7]	87.9% [76.7, 99.0]	90.7% [84.1, 97.3]
2 E:1 T	37	2,942	85%	95%	88.3% [82.3, 94.3]	85.1% [77.0, 93.2]	94.6% [87.3, 100.0]	96.9% [92.7, 100.0]	76.1% [63.8, 88.4]
2 E:2 T	24	1,933	77%	96%	86.5% [79.6, 93.3]	77.1% [65.2, 89.0]	95.8% [90.2, 100.0]	94.9% [87.9, 100.0]	80.7% [70.5, 90.9]
Contact	60	5,124	92%	94%	93.0% [89.2, 96.8]	92.4% [87.0, 97.8]	93.8% [88.4, 99.1]	94.4% [89.7, 99.2]	91.5% [85.4, 97.5]

### 3.4 Label-free temporally consistent apoptosis detection

**FIGURE 8:**
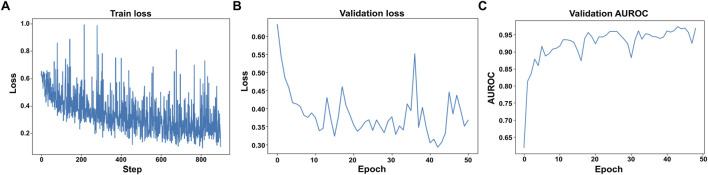
Learning dynamics for label-free temporally consistent apoptosis detection. **(A)** The training loss plotted per optimization step. **(B)** The validation loss plotted per epoch. **(C)** The validation Area Under the ROC Curve (AUROC) plotted per epoch.

The label-free apoptosis model was evaluated on 182 nanowell videos (having 372 cell tracks) whose cell death frames were annotated by the onset of Annexin-V fluorescence and manual verification. A ResNet-18 backbone was selected because the training set is comparatively small and because preliminary experiments showed no benefit from deeper models. During training, the sequence-level loss fell smoothly from ≈0.65 to <0.25, while the validation loss stabilized near 0.33 by epoch 20 and the validation AUROC plateaued at 0.94–0.96 (Figure 8). These curves indicate rapid convergence without over-fitting.

Figure 8 summarizes how the apoptosis classifier learns. In Panel 8.C the AUROC - a measure of how well the model separates dying from live cells, jumps sharply during the first five epochs and then climbs more slowly, showing that the core decision boundary is found early and then fine-tuned. Panel (A) plots the raw training loss for every mini batch; although individual points bounce up and down because of heavy data augmentation, the overall trend drifts steadily downward. Panel (B) tracks the validation loss once per epoch and reveals the same smooth decline, flattening out by about epoch 25. This parallel drop in both training and validation loss indicates that the model is improving without over-fitting.

**FIGURE 9:**
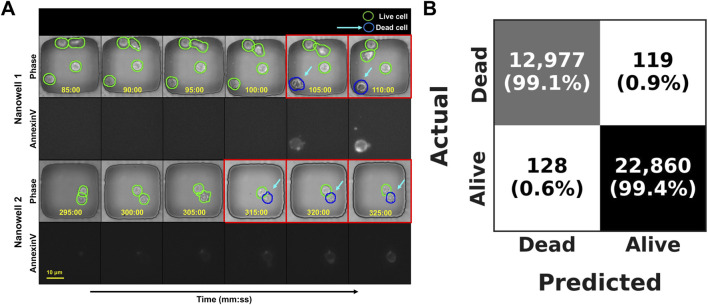
Performance of the Label-Free temporally consistent Apoptosis Detection Module. **(A)** Time-lapse examples from three nanowells, showing the phase-contrast image (top row of each set) and the corresponding monochrome Annexin V fluorescence image (bottom row). The red boxes highlight the time of apoptosis detection. The blue arrows point to cells identified as apoptotic by the model, which aligns precisely with the onset of Annexin V signal (florescent channel), confirming the model’s high temporal accuracy. **(B)** Confusion matrix showing frame-level classification results from 182 nanowells.

We tested the model on 36,084 such cell-patch crops, from the training and validation set, the network was wrong less than one per cent of the time. It flagged 12,977 of the 13,096 truly apoptotic cells (sensitivity = 99.1%) and correctly ignored 22,860 of 22,988 live-cell crops (specificity = 99.5%), for an overall frame-level accuracy of 99.3% and an AUROC of 0.958 (Table 4). At the trajectory level the model was equally precise: for 99.4% of the 372 tracked cells it pin-pointed the death frame to within ±4 imaging frames (≈20 min) of the Annexin-V reference. As illustrated in Figure 9, the detector marks the dying cells (blue arrows) either exactly when Annexin-V first appears or one–two frames earlier, confirming that the cropped phase-contrast texture and brief motion history captured in each patch are sufficient to recognize apoptosis without any fluorescent channel.

**TABLE 4:** Label-Free apoptosis detection metrics.

Metric	Value (%)	95% CI (%)
Accuracy	99.32	[99.23, 99.40]
Sensitivity (recall)	99.09	[98.91, 99.24]
Specificity	99.44	[99.34, 99.53]
Precision (PPV)	99.02	[98.84, 99.18]

## 4 Conclusions and discussion

Our motivation for LF-TIMING is that advancements in immunotherapy lie in comprehensively quantifying cell–cell interactions, which demands dynamic analysis of live cell cohorts at the scale of thousands of cells with minimal cell perturbation. In this context, the spatial confinement provided by the nanowells is crucial since it: (i) enables reliable long-term observation of cell cohorts without the need to follow them over long distances; and (ii) enables reliable quantification of cell behaviors by providing a powerful constraint to the cell tracking algorithms that can be exploited to detect and recover from cell segmentation errors. As noted in our earlier paper (Merouane et al., 2015), the confinement constraint greatly improves the yield of nanowells that are analyzed accurately.

The TIMING assay has previously relied upon fluorescent tagging of cells and markers, and this has limited its ability to access investigative molecular markers of interest. This paper addresses the objective of eliminating the need for fluorescent markers for identification, detection, segmentation, tracking, and identifying cell death, and sets the stage for advancing translational and basic studies by enabling: (1) direct evaluation of low numbers of cells derived from patients being treated with immunotherapies, and (2) the expansion of cellular functionality via the use of fluorescent labels/reporters to study subcellular organelles or cellular metabolism. Our work shows that reliable cell analysis is possible without fluorescent labels by leveraging the phase-contrast channel alone. The deep models are now able to recover per-cell masks and trajectories, quantify intercellular dynamics, and identify cell states such as apoptosis on par with fluorescence based TIMING. The resulting workflow provides automatic, reliable, confinement-constrained image analysis for comprehensive sampling of dynamic cell events and is scalable to multi-terabyte datasets far beyond what manual assessment can provide. Importantly, the LF-TIMING pipeline described here can form the backbone for an integrated AI system that can provide higher-levels of analysis of the cell morphology and dynamics data. We envision that it broadens the application of TIMING as platform to assess the potency of live immune cells wherein cell numbers are limiting like the human post-infusion CAR T cells derived from serial sampling of blood from treated patients.

Our results demonstrate the practicality of a fully label-free TIMING video array analysis pipeline, LF-TIMING, that represents an advance to TIMING analysis because it moves us from fluorescence-constrained observation toward label-free, high-throughput, and biologically generalizable quantification. Much of this advance was made possible by exploiting two powerful constraints–the spatial cell confinement provided by the nanowells, and temporal consistency achieved from analyzing the post-capture nanowell videos at the sequence level rather than at the image frame level. The net result is that the performance of the computational pipeline is comparable to the original fluorescence-based TIMING system. Going forward, we envision even more capable LF-TIMING pipelines being built by leveraging the current pipeline as a scalable foundation for building much larger annotated datasets (Edlund et al., 2021; Pylvänäinen et al., 2023), that, with a modest human-in-the loop proofreading, can enable training ever stronger models. We envision the containment-constrained cell tracker described here to be extended to handle organelle segmentations (Yuan et al., 2019), and handle not only missed cells, but also a variety of other errors arising from optical artifacts, debris, mitosis, fluorescent beads, and other common experimental factors. We also envision novel applications beyond cytotoxicity profiling to large-scale studies of mitosis, organelle dynamics, and cell–cell interaction phenotypes in primary samples. The tools, models, and datasets introduced here open new avenues for minimally perturbative and clinically relevant live-cell analysis at high throughput and yield. These advancements are expected to further improve robustness across TIMING chip designs, imaging conditions, and novel cell types while pushing the system toward fully autonomous, label-free, large-scale, single-cell analytics. In the long run, we expect the LF-TIMING system to form the basis for a clinical translational system that can assess the potency of cell-based infusion products.

## 5 Data sharing and reproducibility

Our segmentation models are based on Mask R-CNN, which is publicly available at https://github.com/facebookresearch/detectron2. Our tracking model is based on the Hungarian algorithm, which is publicly available at https://github.com/tdedecko/hungarian-algorithm. Our cell classifier and apoptosis detection models are based on CNN-LSTM architectures and are publicly available at https://github.com/pranoyr/cnn-lstm. The source code is available to non-profit organizations under an appropriate MTA and can be requested from the corresponding authors. In addition, we have provided sample videos and an interactive interface for segmentation, tracking, and classification at the following website: https://cellchorus.ai/lftiming-manuscript (Username: demo@cellchorus.com, Password: demo123). Along with the sample data, please use the “Manuscript” button on the website to access the videos used in the manuscript.

## Data Availability

The raw data supporting the conclusions of this article will be made available by the authors, without undue reservation.
